# The urate-lowering efficacy and safety of febuxostat in the treatment of the hyperuricemia of gout: the CONFIRMS trial

**DOI:** 10.1186/ar2978

**Published:** 2010-04-06

**Authors:** Michael A Becker, H Ralph Schumacher, Luis R Espinoza, Alvin F Wells, Patricia MacDonald, Eric Lloyd, Christopher Lademacher

**Affiliations:** 1 The University of Chicago Pritzker School of Medicine, MC0930, University of Chicago Medical Center, 5841 S. Maryland Avenue, Chicago, IL 60637, USA; 2 University of Pennsylvania and VA Medical Center, VA Medical Center, 151K University and Woodland Avenues, Philadelphia, PA 19104, USA; 3 Rheumatology, Louisiana State University Health Sciences Center, 2820 Napoleon Avenue, Suite 890, New Orleans, LA 70115, USA; 4 Rosalind Franklin University of Medicine & Science, 1457 Indian Grass Lane, Grays Lake, IL 60030, USA; 5 Takeda Global Research & Development Center Inc., 675 Field Drive, Lake Forest, IL 60045, USA; 6 Astellas Pharma Global Development Inc., 3 Parkway North, Deerfield, IL 60015, USA

## Abstract

**Introduction:**

The purpose of this study was to compare urate-lowering (UL) efficacy and safety of daily febuxostat and allopurinol in subjects with gout and serum urate (sUA) ≥ 8.0 mg/dL in a six-month trial.

**Methods:**

Subjects (n = 2,269) were randomized to febuxostat 40 mg or 80 mg, or allopurinol 300 mg (200 mg in moderate renal impairment). Endpoints included the proportion of all subjects with sUA <6.0 mg/dL and the proportion of subjects with mild/moderate renal impairment and sUA <6.0 mg/dL. Safety assessments included blinded adjudication of each cardiovascular (CV) adverse event (AE) and death.

**Results:**

Comorbidities included: renal impairment (65%); obesity (64%); hyperlipidemia (42%); and hypertension (53%). In febuxostat 40 mg, febuxostat 80 mg, and allopurinol groups, primary endpoint was achieved in 45%, 67%, and 42%, respectively. Febuxostat 40 mg UL was statistically non-inferior to allopurinol, but febuxostat 80 mg was superior to both (*P *< 0.001). Achievement of target sUA in subjects with renal impairment was also superior with febuxostat 80 mg (72%; *P *< 0.001) compared with febuxostat 40 mg (50%) or allopurinol (42%), but febuxostat 40 mg showed greater efficacy than allopurinol (*P *= 0.021). Rates of AEs did not differ across treatment groups. Adjudicated (APTC) CV event rates were 0.0% for febuxostat 40 mg and 0.4% for both febuxostat 80 mg and allopurinol. One death occurred in each febuxostat group and three in the allopurinol group.

**Conclusions:**

Urate-lowering efficacy of febuxostat 80 mg exceeded that of febuxostat 40 mg and allopurinol (300/200 mg), which were comparable. In subjects with mild/moderate renal impairment, both febuxostat doses were more efficacious than allopurinol and equally safe. At the doses tested, safety of febuxostat and allopurinol was comparable.

**Clinical Trial Registration:**

NCT00430248

## Introduction

Gout is an increasingly common disorder [1-5] characterized by hyperuricemia (serum urate concentration (sUA) exceeding 6.8 mg/dL, the limit of urate solubility) and by acute and chronic consequences of urate crystal deposition: episodic gout flares, deforming gouty arthropathy, tophi, and urolithiasis [6]. Long-term management of recurrent or progressive gout focuses on urate-lowering therapy (ULT) aimed at maintaining sUA in a sub-saturating range, often chosen as <6.0 mg/dL [7-12]. Achievement of this goal results in eventual cessation (and even reversal) of urate crystal deposition and clinical signs and symptoms [8-11,13,14]. Pharmacological approaches to ULT have, for over 40 years, involved either enhancement of renal uric acid excretion with a uricosuric, such as probenecid, or inhibition of urate production with the purine base analogue allopurinol, a xanthine oxidase (XO) inhibitor. In the United States, over 90% of ULT prescriptions are for allopurinol, approved at doses up to 800 mg daily but rarely (<5% of patients) prescribed at doses exceeding 300 mg daily [15,16].

Several recent publications [7-11,16-18] have documented poor urate-lowering (UL) efficacy and/or clinical outcomes in gout patients, including progression to gout-related disability and impaired quality of life [7,19-21]. Gout patients also have high incidences of major comorbidities that may be associated with hyperuricemia or gout or both [22]. Thus, gout patients not only suffer potentially disabling and deforming arthritic disease, but are also at high risk for cardiovascular (CV) [23-29] and metabolic disorders [29,30].

Febuxostat, a non-purine analogue XO inhibitor [31], has recently received regulatory approval in the United States for treatment of hyperuricemia in gout patients [32]. Three published randomized controlled trials (RCTs) in subjects with gout and baseline sUA ≥ 8.0 mg/dL [33-35] have established superior UL efficacy (proportion of subjects achieving sUA <6.0 mg/dL) for febuxostat (in daily doses from 80 to 240 mg) compared with placebo [33,35] or with allopurinol 300 mg daily [34,35]. Overall rates of adverse events (AEs) were similar across treatment groups in these studies [33-35], but CV disorders, although uncommon, occurred numerically more often in febuxostat- than allopurinol-treated. Subjects who experienced these events had a prior history of CV disease (CVD) and/or underlying CV risk factors. This imbalance was not statistically significant or explained by known actions of febuxostat but warranted further study.

Here we report results of the CONFIRMS trial, a Phase 3, double-blind RCT further examining the comparative UL efficacy and safety of febuxostat and allopurinol in a greater number of gout subjects than the total participating in prior comparative trials [34,35]. The three-fold objectives of this study were: 1) to compare the UL efficacy of febuxostat at a dose of 40 mg with that of allopurinol at doses most commonly utilized in clinical practice; 2) to evaluate UL efficacies of 80 mg and 40 mg daily doses of febuxostat compared with that of allopurinol in subjects with mild or moderate renal impairment; and 3) to gain prospective, uniformly reviewed information regarding the safety of these agents, particularly their comparative CV safety.

## Materials and methods

### Subjects, study design, and procedures

Subjects aged 18 to 85 years with a diagnosis of gout fulfilling American Rheumatology Association preliminary criteria [36] and sUA ≥ 8.0 mg/dL were eligible for enrollment. Subjects were enrolled at 324 sites in the United States. Institutional Review Board approval was obtained, and all subjects provided written informed consent and Health Insurance Portability and Accountability Act authorization prior to any study-related procedure. At least 35% of subjects enrolled were to have mild or moderate renal impairment, defined as baseline estimated creatinine clearance (eCLcr) of 60 to 89 ml/minutes or 30 to 59 ml/minutes, respectively, calculated by the Cockcroft-Gault formula corrected for ideal body weight [37,38]. Subjects successfully completing either of two previously reported long-term, open-label febuxostat [13] or febuxostat/allopurinol [14] extension studies were also eligible for enrollment.

Exclusion criteria included: secondary hyperuricemia (for example, due to myeloproliferative disorder); xanthinuria; severe renal impairment (eCLcr <30 ml/minutes [37,38]); alanine aminotransferase and aspartate aminotransferase values >1.5 times the upper limit of normal; consumption of more than 14 alcoholic drinks per week or a history of alcoholism or drug abuse within five years; or a medical condition that, in the investigator's opinion, would interfere with treatment, safety, or adherence to the protocol.

Subject screening evaluations included: physical examination and vital signs; medical history, a pre-specified CV history/risk form; laboratory tests (sUA, complete chemistry panel, hematology, urinalysis, and, for women, pregnancy test); echocardiogram; assessment for tophi and gout flare; and concomitant medication use. With the exception of tophus assessment, these elements, along with compliance, were repeated at bimonthly visits during the six-month treatment period. sUA was blinded after baseline determination at Day-4.

An Interactive Voice Response System was utilized by site personnel during screening visits to initiate double-blind randomization. Subjects were randomized 1:1:1 on Day 1 to receive daily febuxostat 40 mg, febuxostat 80 mg, or allopurinol (Apotex; Weston, FL, USA). Randomization was stratified by baseline renal function and prior completion of either of two open-label extension trials [13,14]. Among subjects randomized to allopurinol, those with normal renal function or mild renal impairment received 300 mg daily, and those with moderate renal impairment received 200 mg.

During a 30-day washout period for subjects receiving prior ULT, and throughout the subsequent six-month treatment period for all subjects, prophylaxis for gout flares was given either as colchicine, 0.6 mg daily (West-Ward Pharmaceutical Corporation, Eatontown, NJ, USA) or naproxen, 250 mg twice daily (West-Ward Pharmaceutical Corporation). All subjects receiving naproxen prophylaxis also received lansoprazole 15 mg daily (Takeda Global Research & Development Center, Inc., Deerfield, IL, USA). Choice of prophylaxis regimen was made by the investigator and subject, taking into account prior drug tolerance and prophylaxis experience. In addition, subjects with eCLcr <50 ml/minute were not to receive naproxen. Gout flares were regarded as expected gout manifestations rather than as AEs.

### Efficacy analyses

**Figure 1:**
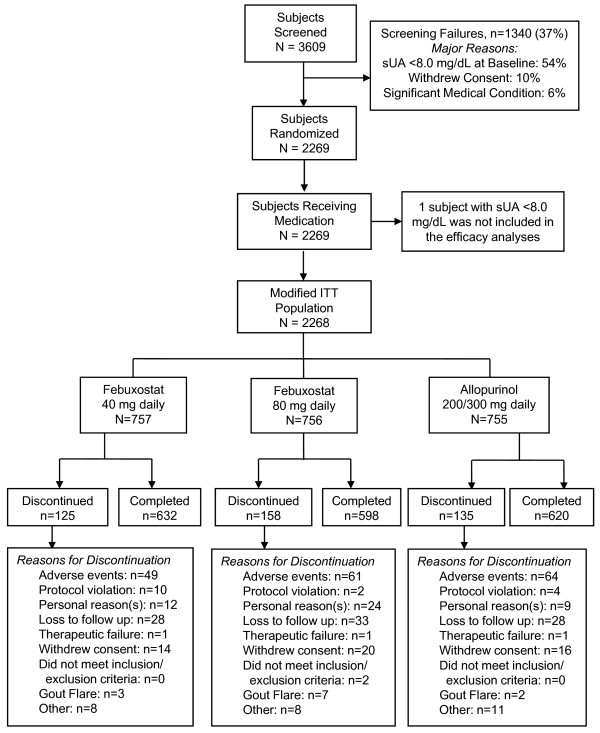
**Flow of subjects through the study**. One subject randomized to allopurinol was not included in the efficacy analyses because baseline serum urate (sUA) did not meet inclusion criterion. The remaining 2,268 subjects eligible for the study comprised the group in which efficacy was assessed.

While 2,269 subjects were randomized to receive at least one dose of febuxostat 40 mg, febuxostat 80 mg, or allopurinol (Figure 1), one subject randomized to the allopurinol treatment group was excluded from the efficacy analyses because baseline sUA was <8.0 mg/dL. Therefore efficacy analyses are based on the 2,268 subjects in the modified intent-to-treat (mITT) cohort.

#### Primary efficacy endpoint

The primary efficacy endpoint was the proportion of subjects in each treatment group with sUA <6.0 mg/dL at the final visit. Prior febuxostat RCTs have demonstrated maintenance of efficacy across last three study visits [34,35]. A major objective of this study was to evaluate the UL efficacy of febuxostat 40 mg; therefore, this treatment group was compared with the allopurinol group with regard to non-inferiority in UL. Binomial 95% confidence intervals (CIs) were calculated for the difference between the two groups in achieving final sUA <6.0 mg/dL. Non-inferiority to allopurinol was to be declared for febuxostat 40 mg if the value of the lower limit of the 95% CI for the difference in achieving the endpoint was greater than -10%. If non-inferiority of febuxostat 40 mg was established, superiority to allopurinol was to be assessed using Fisher's exact test (two-tailed, 0.05 significance level). Efficacy comparisons were also to be made comparing febuxostat 80 mg to allopurinol and to febuxostat 40 mg, using Fisher's exact test (two-tailed, 0.05 significance level).

#### Determination of sample size

A total of 750 subjects per treatment group was required to achieve 90% power to meet the non-inferiority criteria between febuxostat 40 mg and allopurinol for the primary efficacy variable, and to detect a 10% difference between febuxostat 40 mg and allopurinol for the primary efficacy variable, and between febuxostat 40 mg and 80 mg for the primary efficacy variable.

#### Secondary efficacy endpoints

Secondary efficacy variables included: the proportion of subjects with mild or moderate renal impairment and final sUA <6.0 mg/dL; and the proportion of subjects with sUA <6.0 mg/dL, <5.0 mg/dL, and <4.0 mg/dL at each visit. Pairwise comparisons were made between treatment groups with Fisher's exact test.

#### Subgroup analyses of primary endpoint

In subgroup analyses, the primary endpoint was stratified by baseline sUA, renal functional status, presence of tophi at baseline, and prior participation in a ULT trial [13,14]. A separate logistic regression model was fit for each subgroup factor to assess the association between each subgroup and achievement of the sUA endpoint. Each model included treatment, subgroup, and the interaction between treatment and subgroup as explanatory variables.

### Safety analyses

Safety analyses were carried out on the results from all 2,269 subjects. AEs were summarized using Medical Dictionary for Regulatory Activities (MedRA) terminology and reported by count (n) and percentage of subjects reporting any event for that term. Pairwise comparisons between treatment groups were made using Fisher's exact test. As pre-specified, all deaths and AEs considered by investigators to be potentially CV-related were reviewed by a CV Endpoints Committee, composed of three blinded expert adjudicators. The Committee determined if the AE met Antiplatelet Trialists Collaboration (APTC) criteria [39,40] for the following endpoints: CV death, nonfatal myocardial infarction (MI), and nonfatal stroke. Non-APTC CV events, also adjudicated by the Committee, included unstable angina, coronary revascularization, cerebral revascularization, transient ischemic attack, venous and peripheral arterial vascular thrombotic event, congestive heart failure, and arrhythmia.

## Results

### Subject disposition and demographics

Of the 2,269 subjects randomized, 757, 756, and 756 subjects received at least one daily dose of febuxostat 40 mg, febuxostat 80 mg, or allopurinol, respectively (Figure 1). Among allopurinol-treated subjects, 610 received 300 mg daily and 145 subjects received an adjusted dose of 200 mg daily due to moderate renal impairment (eCLcr = 30 to 59 ml/minute). In total, 418 subjects prematurely discontinued treatment, 120 within the first month of treatment.

There were no significant differences across treatment groups in demographic, gout-related, or comorbid characteristics of the subjects enrolled (Table 1). As in prior febuxostat/allopurinol comparative trials [34,35], the study groups were composed largely of white (82%), male (94%), and obese (BMI ≥ 30 kg/m^2^; 64%) persons who acknowledged the use of alcohol (68%). Of the total cohort, 276 had previously completed a three- to five-year open-label ULT trial with either febuxostat or allopurinol [13,14]. These subjects had significantly fewer tophi at baseline compared to those who had not previously participated in either long-term study (12.5% vs 22.3%, respectively; *P *< 0.001); prior participant and non-participant groups did not, however, differ significantly in the proportion of subjects with mild or moderate renal impairment (data not shown).

**Table 1:** Baseline Characteristics of Randomized Subjects^a^

Variable	Febuxostat 40 mg daily N = 757	Febuxostat 80 mg daily N = 756	Allopurinol 200/300 mg daily N = 756
		n (%)	
**Gender**			
Male	722 (95.4)	710 (93.9)	709 (93.8)
Female	35 (4.6)	46 (6.1)	47 (6.2)
			
**Race**			
American Indian or Alaska Native	6 (0.8)	10 (1.3)	6 (0.8)
Asian	26 (3.4)	25 (3.3)	37 (4.9)
Black or African American	83 (11.0)	78 (10.3)	67 (8.9)
Native Hawaiian or Other Pacific			
Islander	11 (1.5)	10 (1.3)	11 (1.5)
White	620 (81.9)	618 (81.7)	625 (82.7)
Other	11 (1.5)	15 (2.0)	8 (1.1)
Missing	0	0	2 (0.3)
			
**Ethnicity**			
Hispanic or Latino	47 (6.2)	49 (6.5)	53 (7.0)
Not Hispanic or Latino	710 (93.8)	707 (93.5)	702 (92.9)
Missing	0	0	1 (0.1)
			
**Age (years)**			
Mean ± SD	52.5 ± 11.68	53.0 ± 11.79	52.9 ± 11.73
Range	21 to 85	21 to 85	19 to 85
			
**Body Mass Index (kg/m^2^)**			
Mean ± SD	32.9 ± 6.37	32.9 ± 6.39	32.7 ± 6.23
Range	20 to 64	16 to 64	17 to 61
			
**Alcohol Use**			
Non-/Ex-drinker	242 (32.0)	243 (32.1)	235 (31.1)
Drinker (1 to 14 drinks/week)	515 (68.0)	513 (67.9)	521 (68.9)
			
**Serum Urate (mg/dL)**			
<9.0	283 (37.4)	280 (37.0)	274 (36.2)
9 to <10.0	225 (29.7)	222 (29.4)	252 (33.3)
10.0 to <11.0	162 (21.4)	159 (21.0)	133 (17.6)
11.0 to <12.0	58 (7.7)	67 (8.9)	64 (8.5)
≥12.0	29 (3.8)	28 (3.7)	33 (4.4)
Mean ± SD	9.6 ± 1.15	9.6 ± 1.20	9.5 ± 1.19
Range	8 to 14	8 to 15	8 to 15
			
**Years with Gout**			
Mean ± SD	12.0 ± 9.13	11.7 ± 9.64	11.2 ± 9.14
Range	0 to 53	0 to 51	0 to 48
			
**Completed Previous Febuxostat Study**	98 (12.9)	88 (11.6)	90 (11.9)
			
**Renal Function^b^**			
Moderately Impaired	130 (17.2)	136 (18.0)	136 (18.0)
Mildly Impaired	349 (46.1)	367 (48.5)	365 (48.3)
Normal	278 (36.7)	253 (33.5)	255 (33.7)
			
**Medical History**			
Cardiovascular Disease (including hypertension)	421 (55.6)	440 (58.2)	436 (57.7)
Diabetes	89 (11.8)	113 (14.9)	110 (14.6)
Hypercholesterolemia	52 (6.9)	53 (7.0)	57 (7.5)
Hyperlipidemia	299 (39.5)	308 (40.7)	335 (44.3)
Use of Low-dose Aspirin (≤ 325 mg daily)	133 (17.6)	133 (17.6)	139 (18.4)

^a^There are no statistically significant differences among treatment groups with respect to the distribution of baseline characteristics.

^b^Moderate baseline renal impairment: estimated creatinine clearance (eCrCl) 30 to 59 ml/minute; mild baseline renal impairment: eCrCl 60 to 89 ml/minute; normal: eCrCl ≥ 90 ml/minute.

Mild or moderate renal impairment was identified in 65% of all subjects. Other comorbid conditions included hypertension (53%), hyperlipidemia (42%), hypercholesterolemia (7%), and diabetes (14%). The majority of subjects (87%) reported use of concomitant medications, including medications acting on the renin-angiotensin system (36%), lipid-modifying agents (31%), and anti-inflammatory and anti-rheumatic products (29%). Greater than 90% adherence to treatment was recorded for 80% of the subjects.

### Efficacy analyses

#### Primary efficacy endpoint

The proportions of subjects achieving a final visit sUA <6.0 mg/dL, were 45.2%, 67.1%, and 42.1% in the febuxostat 40 mg, febuxostat 80 mg, and allopurinol groups, respectively. UL by febuxostat 40 mg was non-inferior to that by allopurinol: but the difference in the response rates between the two groups (3.1%, 95% CI: -1.9% to 8.1%) was not significant. However, the greater UL response rate with febuxostat 80 mg compared with either febuxostat 40 mg (21.9%) or allopurinol (24.9%) was significant (*P *< 0.001).

#### Secondary efficacy endpoints

Among subjects with any (mild or moderate) renal impairment, the UL response rate in the febuxostat 80 mg group (71.6%; 360/503) significantly exceeded those observed in the febuxostat 40 mg (49.7%; 238/479) and allopurinol (42.3%; 212/501) groups (*P *≤ 0.001, for each comparison). In addition, among the total group of subjects with renal impairment, the UL response rate in the febuxostat 40 mg group was significantly higher than that in the allopurinol 300/200 mg group (*P *= 0.021).

At any sUA endpoint (<6.0 mg/dL, <5.0 mg/dL, or <4.0 mg/dL) and at any scheduled visit, a significantly higher proportion of subjects receiving febuxostat 80 mg achieved the target endpoint than subjects receiving febuxostat 40 mg or allopurinol (*P *< 0.001). Greater proportions of subjects in the febuxostat 40 mg group than the allopurinol group achieved sUA <6.0 mg/dL (at Month 2 visit; *P *= 0.031) and <5.0 mg/dL (at two- and six-month visits; *P *≤ 0.05), but significant differences were not seen at other visits, including the final visit. There were no differences between febuxostat 40 mg and allopurinol groups in achievement of sUA <4.0 mg/dL.

#### Subgroup analyses of the primary endpoint

Among baseline characteristics, higher sUA, presence of tophi, and impaired renal status significantly (*P *< 0.001) and independently affected achievement of the primary endpoint: higher sUA and tophi were associated with lower rates of endpoint achievement, and subjects with mild renal impairment had uniformly higher rates of reaching goal sUA than subjects with normal renal function (Table 2).

**Table 2:** Effect of baseline characteristics on treatment response represented as the percentage of mITT subjects in each subgroup achieving sUA <6.0 mg/dL at Final Visit

Variable	Febuxostat 40 mg daily N = 757	Febuxostat 80 mg daily N = 756	Allopurinol 300/200 mg daily N = 755
		% (n/N)	
**Renal Function^a^**			
Moderately Impaired	43.1 (56/130)	71.3 (97/136)	31.6 (43/136)
Mildly Impaired	52.1 (182/349)	71.7 (263/367)	46.3 (169/365)
Normal	37.4 (104/278)	58.1 (147/253)	41.7 (106/254)
			
**Baseline Serum Urate (mg/dL)^a^**			
<9.0	60.1 (170/283)	80.4 (225/280)	52.7 (144/273)
9.0 to <10.0	47.1 (106/225)	70.7 (157/222)	40.5 (102/252)
≥10.0	26.5 (66/249)	49.2 (125/254)	31.3 (72/230)
			
**Baseline Tophus^a^**			
No	48.1 (284/591)	69.8 (414/593)	44.6 (271/607)
Yes	34.9 (58/166)	57.1 (93/163)	31.8 (47/148)
			
**Completed Prior Febuxostat Study^a^**			
No	43.4 (286/659)	65.7 (439/668)	40.8 (271/665)
Yes	57.1 (56/98)	77.3 (68/88)	52.2 (47/90)

^a^Variable had a significant (*P *< 0.001) overall effect on attainment of the primary endpoint. After adjusting for the variable, febuxostat 80 mg remained statistically significant compared to both febuxostat 40 mg and allopurinol.

Among the 276 subjects who had maintained long-term goal range sUA over three to five years in prior ULT trials [13,14], sUA <6.0 mg/dL was achieved at the final visit in 57.1%, 77.3%, and 52.2%, respectively, of subjects assigned to the febuxostat 40 mg, febuxostat 80 mg, and allopurinol groups (Table 2). Achievement of primary endpoint in subjects not previously so treated were, respectively, 43.4%, 65.7%, and 40.8% (*P *≤ 0.05 for comparison of subjects with and without prior participation for each treatment group).

#### Gout flares

**Figure 2:**
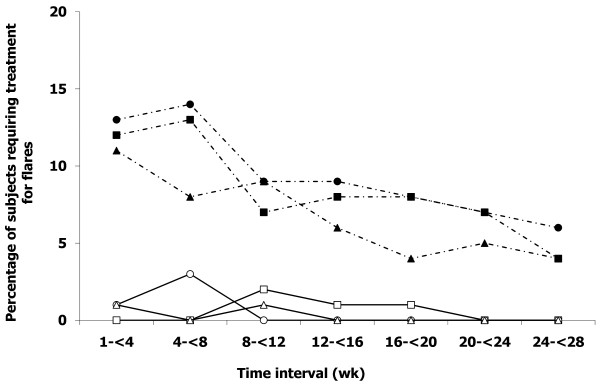
**Proportion of subjects requiring gout flare treatment**. Solid lines, open symbols, prior participation in long-term open-label ULT extension trials [13,14]. Dashed lines and closed symbols, no prior participation in long-term open-label ULT extension trials; squares, febuxostat 40 mg daily; circles, febuxostat 80 mg daily; triangles, allopurinol 200/300 mg daily.

Rates of flare requiring treatment occurred in 10% to 15% of subjects in all treatment groups during each of the first two months of treatment but declined slowly over the subsequent course of the trial (Figure 2). Of particular note, subjects with prior long-term ULT [13,14] in each treatment group in CONFIRMS had lower rates of flares requiring treatment compared to subjects in the corresponding group without prior participation (*P *≤ 0.001 for each comparison).

### Safety Analyses

#### Rates of AEs and premature withdrawals

AEs were reported by 56% of subjects (1,272/2,269). Occurrence rates did not differ among treatment groups (Table 3), nor did rates of most frequently reported AEs. The majority of AEs were mild or moderate in severity.

**Table 3:** Adverse events

	Febuxostat 40 mg daily N = 757	Febuxostat 80 mg daily N = 756 n (%)	Allopurinol 200/300 mg daily N = 756
Total Subjects with ≥ 1 AE	429 (56.7)	410 (54.2)	433 (57.3)
			
Total Subjects With at ≥ 1 Serious AE	19 (2.5)	28 (3.7)	31 (4.1)
			
**Most Frequently (≥ 5%) Reported AEs**			
Upper Respiratory Tract Infections	71 (9.4)	53 (7.0)	57 (7.5)
Liver Function Analysis	63 (8.3)	52 (6.9)	50 (6.6)
Rash	44 (5.8)	42 (5.6)	55 (7.3)
Diarrhea	45 (5.9)	47 (6.2)	57 (7.5)
Musculoskeletal and Connective Tissue Signs and Symptoms NEC	43 (5.7)	38 (5.0)	32 (4.2)
			
**All APTC CV Events**			
Number of Subjects with Events	0	3	3
Rate (%)	0.00	0.40	0.40
95% CI (%)	(0.000, 0.486)	(0.082, 1.155)	(0.082, 1.155)
			
**APTC Events Summarized by Category**			
CV Death	0	0	2 (0.26)
Nonfatal Myocardial Infarction	0	1 (0.13)	1 (0.13)
Nonfatal Stroke	0	2 (0.26)	0
			
**All Non-APTC Events**			
Number of Subjects with Events	10	9	7
Rate (%)	1.32	1.19	0.93
95% CI (%)	(0.635, 2.416)	(0.546, 2.248)	(0.373, 1.898)
			
**Non-APTC Events Summarized by Category**			
Angina	2 (0.26)	0	0
Coronary Revascularization	1 (0.13)	0	1 (0.13)
Transient Ischemic Attack	1 (0.13)	0	1 (0.13)
Cerebral Revascularization	0	0	0
Venous and Peripheral Arterial Vascular Thrombotic Event	0	2 (0.26)	0
Congestive Heart Failure	2 (0.26)	0	1 (0.13)
Arrhythmia, No Evidence of Ischemia	3 (0.40)	4 (0.53)	1 (0.13)
Other Non-APTC CV Events	1 (0.13)	3 (0.40)	3 (0.40)

AE: adverse events; APTC: antiplatelet trialists collaboration; CI: confidence interval; CV: cardiovascular; NEC: not elsewhere characterized

Rates of AEs in subjects with mild or moderate renal impairment were similar to those in the overall study population: 56% (268/479), 54% (270/503), and 58% (289/501) of subjects in the febuxostat 40 mg, febuxostat 80 mg, and allopurinol groups, respectively, reported at least one AE. The most frequently reported AEs in subjects with renal impairment were the same as those reported for all subjects. Although not significant, rates of abnormal liver function tests were lower among subjects with mild (6% to 7%) or moderate renal impairment (3% to 6%) compared to subjects with normal renal function (9% to 10%) in all treatment groups. Rates of diarrhea were higher among subjects with moderate renal impairment receiving febuxostat (8% to 10%), compared with subjects with moderate renal impairment receiving allopurinol (7%).

Most frequently reported serious AEs (>0.1% of all subjects) were non-specific bacterial infections, coronary artery disease, ischemic coronary artery disorders, lower respiratory tract infections, and prostate cancer. Five subjects died during the study: one subject each receiving febuxostat 40 mg (anaphylactic reaction after 50 fire ant stings) and febuxostat 80 mg (brain edema and chronic obstructive pulmonary disease); and three subjects receiving allopurinol (hypertensive heart disease; necrotizing pneumonia and sepsis after surgery for lung adenocarcinoma; and sudden death). No deaths were judged by investigators to be related to a study drug.

Differences among treatment groups in premature discontinuation rates due to AEs occurring during the study were not significant and are detailed in Figure 1. The most common AEs leading to discontinuation were investigator-defined liver function abnormalities (reported in 2%, 1%, and 1% of subjects receiving febuxostat 40 mg, febuxostat 80 mg, and allopurinol, respectively).

#### AEs in specific organ systems

Rash AEs were reported by 6%, 6%, and 7% of subjects receiving febuxostat 40 mg, febuxostat 80 mg, and allopurinol, respectively. The most frequently reported rash AEs were dermatitis and eczema (2%, 2%, and 3%, respectively) and rashes, eruptions, and exanthems (2%, 2%, and 1%, respectively). With the exception of contact dermatitis, no specific rash was experienced by >1% of subjects in any treatment group, and differences in incidence between groups were not significant. Eighteen subjects withdrew prematurely due to a rash AE, including one subject, receiving allopurinol, who had a severe desquamating eruption requiring parenteral corticosteroid therapy.

Rates of liver function abnormalities are shown in Table 3. Aminotransferase elevations were generally mild (not exceeding three-fold the upper limit of normal), transient even with continued study drug treatment, and of similar frequency in febuxostat- and allopurinol-treated subjects. No subjects with combined transaminase increases and serum bilirubin levels exceeding twice the upper limit of normal were observed. Among subjects taking colchicine for flare prophylaxis, the incidence of increased transaminases was similar across treatment groups.

#### Cardiovascular AEs

At least one CV AE was investigator-reported in 5% of all subjects: 5%, 5%, and 6% of subjects in the febuxostat 40 mg, febuxostat 80 mg, and allopurinol groups, respectively. No difference between treatment groups in specific CV AEs was detected. Pre-specified adjudication of all deaths and all AEs reported to be CV system-related identified a total of six subjects experiencing an adjudicated APTC event: three in the febuxostat 80 mg group and three in the allopurinol group (Table 3). All subjects experiencing an adjudicated APTC event had prior medical histories of or underlying risk factors for CVD. Differences in the rates of adjudicated non-APTC CV events between treatment groups were not significant.

## Discussion

In a previous RCT in subjects with gout [33], the UL efficacy of febuxostat at a daily dose of 40 mg was superior to that of placebo. Here, the UL efficacy of febuxostat 40 mg was confirmed as comparable to that of allopurinol 300 mg. At all levels of renal function studied, daily dosing of febuxostat 80 mg was superior in UL efficacy to that of febuxostat 40 mg or allopurinol. However, in subjects with impaired renal function, febuxostat 40 mg was also superior to allopurinol 300 mg daily (mild renal impairment) or 200 mg daily (moderate renal impairment), suggesting that treatment with either dose of febuxostat is more likely to achieve sUA <6.0 mg/dL than is allopurinol treatment at the reduced doses widely recommended for such individuals [41]. Indicators of higher body urate pools (baseline presence of tophi or sUA ≥ 10.0 mg/dL) were also identified as factors reducing rates of responsiveness to ULT.

If goal range urate levels are not achieved and maintained with febuxostat 40 mg daily or allopurinol, the current data suggest that dose titration to febuxostat 80 mg would be a rational alternative to increasing allopurinol doses beyond 300 mg. In fact, RCT evidence to support the safety and efficacy of allopurinol administered at doses exceeding 300 mg daily is limited to a recent four-month trial in which 13 of 17 subjects (eCLcr ≥ 50 ml/minute) failing to achieve sUA <5.0 mg/dL while receiving allopurinol 300 mg daily safely reached this efficacy endpoint when treated with 600 mg daily [42].

Failure to achieve sUA <6.0 mg/dL among individuals with or without renal impairment receiving guideline-recommended doses of allopurinol has been documented [43]. The common practice of prescribing allopurinol at a fixed daily dose of 300 mg for gout patients with mildly impaired or normal renal function and less for those with moderate renal impairment [12,18,44] may further decrease the likelihood of achieving sUA <6.0 mg/dL. In this study, there was no titration of allopurinol doses; subjects were assigned the standard daily dose of 300 mg unless they had moderate renal impairment. For this reason and due to the paucity of controlled data cited above [42], we are reluctant to speculate as to whether allopurinol dose escalation would have safely increased the proportion of subjects who achieved target sUA [42,45].

The prior RCTs comparing febuxostat and allopurinol [34,35] did not establish significant differences between these agents in the clinical outcomes of flare rate and tophus size reductions in the first 6 to 12 months of treatment. As such, clinical outcomes were not endpoints in the current trial. Nevertheless, subjects who had successfully completed prior the long-term open-label UL studies [13,14] and then enrolled in CONFIRMS had reduced rates of baseline tophi and, during the additional six months of ULT, showed higher rates of UL efficacy and reduced rates of gout flares compared with those who did not have prior trial participation. These data support the concept that persistent UL to sub-saturating sUA eventually results in relief of clinical symptoms of both acute (flares) and chronic (tophi) gout [8,10,12] likely by depletion of excessive miscible and non-miscible (crystalline) extracellular urate pools characteristic of gout patients [46,47].

In all treatment groups, rates of gout flares requiring treatment diminished slowly after Month 2 and did not affect more than 15% of subjects per month after Week 8 of the study (Figure 2). This result contrasts with sharp increases in flare rates (30% to 40% of subjects per month) encountered after cessation of flare prophylaxis at Month 2 in prior comparative RCTs [34,35]. These results seem likely due to differences in the duration of prophylaxis and are in accord with the results of an RCT that established the efficacy of colchicine flare prophylaxis during the first six months after initiation of allopurinol therapy [48]. We thus recommend gout flare prophylaxis co-therapy for at least the first six months of ULT.

As in prior febuxostat clinical trials [13,14,33-35], no severe rashes or hypersensitivity reactions were observed during febuxostat treatment in the current trial. In these trials, serum transaminase elevations (≥three times upper limit of normal) were numerically more frequent with febuxostat than with allopurinol, but neither concurrent serum bilirubin levels exceeding 2 mg/dL nor clinically-manifested liver-related symptoms were encountered [34,35]. In the entire febuxostat development program [13,14,33-35], neither febuxostat nor allopurinol treatment was associated with combined serum transaminase and bilirubin increases, except when explained by cholecystitis or by bile duct obstruction due to stones or malignancy. Nevertheless, administration of allopurinol or febuxostat to a gout population with significant rates of obesity, metabolic syndrome, and alcohol use [34,35] is accompanied by liver function test abnormalities more frequently than is placebo administration [35]. Consequently, we recommend periodic liver function testing during treatment with either febuxostat or allopurinol.

A lack of significant differences in overall rates of AEs or of the most frequently occurring treatment emergent AEs across febuxostat and allopurinol treatment groups in CONFIRMS recapitulate safety results previously reported [34,35]. In addition, in this study encompassing a greater number of subjects than all prior febuxostat/allopurinol RCTs combined, no significant differences between treatment groups were observed in the rates of reported CV AEs, adjudicated APTC, or non-APTC events. Gout and hyperuricemia are strongly associated with CVD and CVD-related death [24-29,49], and all subjects who experienced adjudicated CV events had prior medical histories or risk factors for CVD. Thus, despite the fact that the small number of severe CV AEs encountered in febuxostat/allopurinol comparative RCTs did not identify a differential CV risk of these agents, we recommend continued attention to monitoring and management of CV health, particularly among gout patients at high risk for CV events.

## Conclusions

Results of the CONFIRMS trial establish equivalent UL efficacy for febuxostat 40 mg daily and allopurinol 300/200 mg daily. At all levels of renal function studied, UL efficacy of febuxostat 80 mg daily was superior to that of febuxostat 40 mg or allopurinol 300/200 mg, and was comparably safe. In subjects with mildly or moderately impaired renal function, however, febuxostat 40 mg daily was significantly more effective in lowering sUA than allopurinol. At the doses studied, safety of febuxostat and allopurinol, including CV safety, was comparable. Febuxostat, at 40 mg or 80 mg daily, offers a well-tolerated UL alternative to allopurinol, particularly for patients with mild or moderate renal impairment.

## Abbreviations

AE: adverse event; APTC: Antiplatelet Trialists Collaboration; BMI: body mass index; CI: confidence interval; CV: cardiovascular; CVD: cardiovascular disease; eCLcr: estimated creatinine clearance; mITT: modified intent-to-treat; RCT: randomized controlled trial; sUA: serum urate; UL: urate lowering; ULT: urate-lowering therapy; XO: xanthine oxidase.

## Competing interests

MAB has served as a consultant for Takeda Global Research & Development Center, Inc. and Savient Pharmaceuticals (>$10,000) and for Biocryst Pharmaceuticals, Regeneron Pharmaceuticals, Proctor & Gamble, Inc., and URL Pharma (<$10,000). LRE has received consulting fees from Takeda Global Research & Development Center, Inc. (<$10,000). HRS has received consulting fees (<$10,000) from Takeda Global Research & Development Center, Inc., Savient Pharmaceuticals, Regeneron Pharmaceuticals, Pfizer, Inc., Xoma, Centocor, Inc., and has received research grants from Takeda Global Research & Development Center, Inc., CherryPharm, and Wyeth Pharmaceuticals (now a part of Pfizer, Inc.). AFW has served as a consultant for Takeda Global Research & Development Center, Inc.(>$10,000), Abbott Laboratories, Amgen, Inc., Bristol-Myers Squibb Company, Centocor, Inc., Eli Lilly and Company, GlaxoSmithKline, Pfizer, Inc., Wyeth Pharmaceuticals, and Genetech, Inc. (all <$10,000). PM and EL are currently employees of Takeda Global Research & Development Center, Inc., Deerfield, IL, USA. They were employees of TAP Pharmaceutical Products Inc. at the time of the study conduct and analysis. CL was an employee of TAP Pharmaceutical Products Inc. at the time of the study conduct and analysis.

## Authors' contributions

MAB and PM made substantial contributions to the conception and design of the study; acquisition, analysis and interpretation of the data; drafting and revising of the manuscript; and gave final approval for publication. HRS participated in the analysis and interpretation of the data; drafting and revising of the manuscript; and gave final approval for publication. LRE and AFW were involved in drafting and revising the manuscript; and gave final approval for publication. EL and CL contributed to the conception and design of the study; analysis and interpretation of the data; drafting and revising of the manuscript; and gave final approval for publication.
